# The Influence of Sildenafil–Metformin Combination on Hyperalgesia and Biochemical Markers in Diabetic Neuropathy in Mice

**DOI:** 10.3390/medicina59081375

**Published:** 2023-07-27

**Authors:** Ciprian Pușcașu, Anca Ungurianu, Oana Cristina Șeremet, Corina Andrei, Dragoș Paul Mihai, Simona Negreș

**Affiliations:** Faculty of Pharmacy, “Carol Davila” University of Medicine and Pharmacy, Traian Vuia 6, 020956 Bucharest, Romania; ciprian.puscasu@umfcd.ro (C.P.); simona.negres@umfcd.ro (S.N.)

**Keywords:** diabetic neuropathy, sildenafil, metformin, combination, antihyperalgesic

## Abstract

*Background and objectives*: Worldwide, approximately 500 million people suffer from diabetes and at least 50% of these people develop neuropathy. Currently, therapeutic strategies for reducing diabetic neuropathy (DN)-associated pain are limited and have several side effects. The purpose of the study was to evaluate the antihyperalgesic action of different sildenafil (phosphodiesterase-5 inhibitor) and metformin (antihyperglycemic agent) combinations in alloxan-induced DN. *Methods*: The study included 100 diabetic mice and 20 non-diabetic mice that were subjected to hot and cold stimulus tests. Furthermore, we determined the influence of this combination on TNF-α, IL-6 and nitrites levels in brain and liver tissues. *Results*: In both the hot-plate and tail withdrawal test, all sildenafil–metformin combinations administered in our study showed a significant increase in pain reaction latencies when compared to the diabetic control group. Furthermore, all combinations decreased blood glucose levels due to the hypoglycemic effect of metformin. Additionally, changes in nitrite levels and pro-inflammatory cytokines (TNF-α and IL-6) were observed after 14 days of treatment with different sildenafil–metformin combinations. *Conclusions*: The combination of these two substances increased the pain reaction latency of diabetic animals in a dose-dependent manner. Moreover, all sildenafil–metformin combinations significantly reduced the concentration of nitrites in the brain and liver, which are final products formed under the action of iNOS.

## 1. Introduction

According to the World Health Organization, diabetes is “a chronic metabolic disease, characterized by increased levels of glucose in the blood, which leads, over time, to serious conditions of the nerves, kidneys, heart, blood vessels and eyes”. Diabetes causes abnormalities in carbohydrate, lipid, and protein metabolism, and its clinical signs include polydipsia, polyphagia, polyuria, weight loss, and blurred vision [1].

Diabetic neuropathy (DN) has the highest incidence of all forms of neuropathy. Worldwide, The International Diabetes Federation approximates that 537 million people suffer from diabetes [2], and of these, at least 50% develop DN over time [3]. People with type-2 diabetes have a higher incidence of neuropathy than people with type-1 diabetes. Moreover, for type-2 diabetes patients diagnosed for more than 10 years, the prevalence of diabetic neuropathy increases from 8% to 42% [3]. According to a large study that included 6500 participants, 28.5% of them had diabetic neuropathy [4]. Another study comprising 15,000 diabetic individuals pointed out that 35% had painful neuropathy, with women and South Asians having an increased risk of developing symptoms of neuropathy [5].

This pathology varies from asymptomatic to motor, sensory, and vegetative nerve dysfunctions [6]. Once complications have occurred, DN is difficult to treat, and patients face a high risk of amputations associated with increased mortality [7,8,9,10]. The mechanisms involved in the development of DN are not fully elucidated. Peripheral nerve microvascular changes, metabolic and autoimmune disturbances accompanied by glial cell activation, changes in sodium and calcium channel expression, and, more recently, central pain mechanisms such as increased thalamic vascularity and imbalance of descending facilitatory/inhibitory pathways are all mechanisms that contribute to the development of this pathology [11,12].

It is well-known that TNF-α (tumor necrosis factor-α) is involved in the pathophysiology of chronic pain [13]. Additionally, an inflammatory process occurs at the level of nerve tissues in diabetic patients, and several studies showed an increase in blood pro-inflammatory cytokines [14,15]. Besides TNF-α, IL-6 (interleukin-6) production undergoes an increase in diabetic patients with neuropathy [16].

iNOS (inducible nitric oxide synthase) is a pro-inflammatory enzyme that generates intracellular free radicals and increases NO (nitric oxide) production [17]. Consequently, NO contributes to the progression of neuropathic pain by directly affecting injured peripheral axons and acting as a signaling molecule in the medullary dorsal horn [18].

Considering the diversity of mechanisms implicated in the occurrence of DN, there is a great need for new therapeutic options with improved efficacy in treating this pathology. Currently, therapeutic strategies have limited success in reducing pain, as well as a high risk of adverse reactions [19]. Tricyclic antidepressants (amitriptyline), calcium channel blockers (gabapentin, pregabalin), and serotonin and norepinephrine reuptake inhibitors (duloxetine, venlafaxine) are recommended as first-line treatments according to the American Academy of Neurology (AAN) [20] and European Federation of Neurological Society (EFNS) [21]. As a second-line therapy, tramadol can be used and when a topical treatment is preferred, capsaicin can be administered. Strong opioids are recommended as third-line treatments due to their high risk of abuse and the fact that they have not demonstrated their superiority compared to non-opioids [20,21].

Previous studies have highlighted the ability of sildenafil, a PDE5 (phosphodiesterase-5) inhibitor, to alleviate DN-associated pain via significantly increasing the number of functional blood vessels in the sciatic nerve and improving neurovascular function [22]. Wang et al. showed that the administration of sildenafil to 36-week-old mice decreases hypersensitivity to a thermal stimulus, enhances conduction in the sciatic nerve, and increases sciatic nerve myelin thickness [22].

On the other hand, metformin, an antihyperglycemic agent, prevented the installation of peripheral neuropathy by activating AMP-activated protein kinase (AMPK), which is considered a target for pain treatment because it regulates a diversity of cellular processes involved in chronic pain by producing changes in the function and phenotype of peripheral nociceptive neurons [23]. Byrne et al. demonstrated that metformin reduced hyperalgesia in rats treated with fructose, which suggests that its antihyperalgesic effect is not entirely dependent on its hypoglycemic action [24]. In addition, Deftu et al. highlighted that metformin activates AMPK, which in consequence modulates the activity of the E3 ubiquitin ligase NEDD4-2 and the expression of voltage-gated sodium channels (NaVs) [25].

Our study aimed to investigate the antihyperalgesic effect of a sildenafil–metformin combination for the first time in an alloxan-induced diabetic-neuropathy model and its influence on inflammation (via pro-inflammatory cytokines and nitrites). In a previous study, we demonstrated that metformin and sildenafil administered individually can reduce thermal hyperalgesia after 14 consecutive days of treatment [26], but recent findings have shown that a single-drug approach to chronic disease is characterized by limited efficacy, many side effects, and the development of resistance to treatment. Furthermore, the results of several studies highlighted that combined therapy is a more efficient option to treat chronic diseases with complex etiology and pathophysiology such as diabetes, cancer, or AIDS [27,28,29]. Current guidelines recommend, in the majority of situations, monotherapy in diabetic neuropathy, but the efficacy and safety are frequently limited [30,31]. As a consequence, the therapeutic approach to diabetic neuropathy with a combination of two drugs that ameliorate pain through different mechanisms of action could be a better option and could overcome these limitations.

## 2. Materials and Methods

### 2.1. Reagents

Alloxan was purchased from Sigma Chemical Company (Hamburg, Germany, product no. A7413), sildenafil was purchased from Actavis Group PTC EHF (Hafnarfirdi, Island), metformin was purchased from Gedeon Richter PLC (Budapest, Hungary), gabapentin was purchased from Egis Pharmaceuticals PLC (Budapest, Hungary), and thiopental sodium was purchased from Sigma-Aldrich (St. Louis, MO, USA).

### 2.2. Experimental Animals

The bioethics norms for research on experimental animals for scientific purposes proposed by Law 43/2014 on the protection of experimental animals and Directive 2010/63/EU of the European Parliament on the protection of animals used for experimental carried out were herein respected. The experimental protocol was approved by the Bioethics Committee of the Faculty of Pharmacy, Carol Davila University of Medicine and Pharmacy, Bucharest, Romania (CFF38/05.12.2022).

Male NMRI mice (4–8 weeks old) were acquired from INCDMI Cantacuzino (Cantacuzino National Institute of Research, Bucharest, Romania). The mice used in the experiment were placed in plexiglass cages and had access to food (rodent ground chow, INCDMI Cantacuzino, Bucharest, Romania) and water ad libitum. The temperature and humidity of the environment, which were assessed with the help of a hygrothermometer, were maintained during the experiment at 21–24 °C and 45–60%, respectively.

### 2.3. Induction of Diabetes Mellitus

The mice (n = 200, 25 ± 2.5 g) were left to acclimatize to the new habitat for one week before the induction of diabetes with alloxan 150 mg·kg^−1^ i.p. (intraperitoneally). Before the administration of alloxan, the animals were fasted for 24 h. The blood sugar of the animals was determined 48 h afterward with an ACCU-CHEK Active glucometer (Roche Diagnostics GmbH, D-68298 Mannheim, Germany) by collecting blood from the veins of the tail (puncture). The diabetic animals, with blood glucose values ≥180 mg/dL, were selected for further testing. If the blood sugar was lower than 180 mg/dL, the mice received one or two doses of alloxan (150 mg·kg^−1^ i.p., at 48 h intervals) and were selected if they became diabetic [26,30]. The determination was made in the morning at 8:00 AM under fasting conditions for 24 h before the determination. For this study, we used 100 diabetic mice and 20 non-diabetic mice divided into equal groups (n = 20) that received treatments daily, for 14 days by oral gavage, as follows: **ND** group—non-diabetic control group that received distilled water 0.1 mL·kg^−1^; **D** group—diabetic control group that received distilled water 0.1 mL·kg^−1^; **G** group—gabapentin 100 mg·kg^−1^; **S1.5 + M150** group—sildenafil 1.5 mg·kg^−1^ + metformin 150 mg·kg^−1^; **S2.5 + M250** group—sildenafil 2.5 mg·kg^−1^ + metformin 250 mg·kg^−1^; **S3 + M500** group—sildenafil 3 mg·kg^−1^ + metformin 500 mg·kg^−1^.

### 2.4. Blood Glucose Levels

The blood glucose (mg/dL) was determined at the initial moment and 7 and 14 days after the groups were formed, with the ACCU-CHEK Active device by collecting blood from the veins of the tail (puncture).

### 2.5. Tests for the Evaluation of the Antihyperalgesic Effect

#### 2.5.1. Heat Hypersensitivity

Thermal hypersensitivity was assessed using the hot-plate test at the initial moment and 7 and 14 days from the beginning of treatment. The mice were placed on a plate heated to 53 °C and the elapsed time was recorded until the first sign of thermal sensitivity appeared: licking, jumping, or shaking of the paws [32].

#### 2.5.2. Cold Allodynia

The sensitivity to a cold thermal stimulus was tested by the tail withdrawal test. Each mouse tail was immersed by halfway through length into water cooled to 10 ± 0.5 °C maintained with the help of an ice bath and the time elapsed from the immersion of the tail in the water to its withdrawal was recorded. In all cases, a maximum of 25 s was maintained to avoid the appearance of tail injuries. The determination was made initially and 7 and 14 days after the administration of substances [33,34].

### 2.6. Biochemical Assay of Mouse Brain and Liver Homogenates

Mouse brains and livers were harvested after euthanasia with 200 mg·kg^−1^ of thiopental sodium i.p. [35]. For tissue homogenates, a 1:10 ratio (*w*/*v*) between tissue and PBS (Biochrom AG, Berlin, Germany) was used and then homogenized with an RW 14 basic homogenizer (IKA, Staufen, Germany) and diluted 1:10 with PBS prior to experimental procedures.

#### 2.6.1. Assessment of TNF-α and Il-6

TNF-α (catalog no. LS-F5192-1) and Il-6 (catalog no. LS-F11810) were assessed according to the manual guide contained in the kit from LSBio (LifeSpan BioSciences, Inc., Seattle, WA, USA).

#### 2.6.2. Griess Assessment of NOS Activity

With the aid of the previously modified employed Griess method [36] (vanadium (III) was used to reduce nitrates to nitrites [37]), we determined brain and liver nitrites as the end-products of nitric oxide synthase (NOS). Thus, after tissue homogenate was treated with Griess modified reagent 4% (100 µL, Sigma Aldrich, St. Louis, MO, USA, product no. G4410), OD (optical density) was read at 540 nm after incubation for 5 min at room temperature. A standard curve of NaNO_2_ (Sigma Aldrich, St. Louis, MO, USA, product no. 67398) was measured and results are reported as µM NO_2_-equivalents to protein ratio. For each sample, a blank was read and the necessary corrections were made.

#### 2.6.3. Protein Content

Total protein content in tissue homogenates and mitochondrial preparations was evaluated using the well-established Lowry method [38] with a standard curve of bovine serum albumin up to 1.5 mg/mL.

### 2.7. Statistical Analysis

The experimental data obtained were statistically analyzed using the GraphPad Prism v.5.00 software package (GraphPad Software, San Diego, CA, USA). D’Agostino-A Pearson test was applied to determine the type of data distribution. For parametric data, the one-way analysis of variance (ANOVA) test followed by Dunnet’s post hoc test were used, while for nonparametric data Kruskal–Wallis test followed by Dunn’s post hoc test were applied to analyze the experimental results. The level of statistical significance was α = 0.05, the confidence interval for the statistical tests performed was 95%, and the experimental results were expressed as individual mean values ± standard error of the mean (S.E.M.). The following Formula (1) was applied to calculate the percentage variations of experimental data between groups [39]:

Δ% = (Mx − My)/My × 100(1)

where, Mx is the mean value for D when compared vs. ND, or GBN, S1.5 + M150, S2.5 + M250, and S3 + M500 groups when compared vs. D; My is the mean value for either ND or D.

## 3. Results

### 3.1. Blood Glucose Level

**Figure 1:**
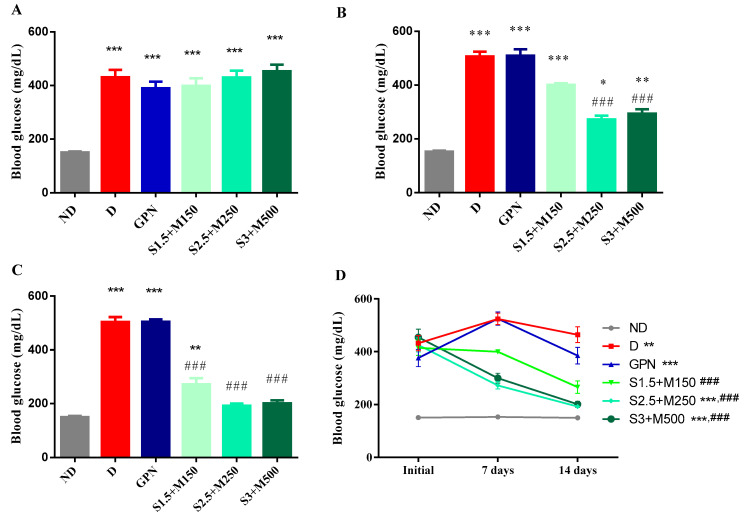
(**A**) Initial blood glucose values (**B**). Blood glucose values at 7 days (**C**). Blood glucose values at 14 days. (**D**) Evolution of the mean blood glucose levels during the experiment. Values are expressed as mean ± S.E.M. ND—non-diabetic control; D—diabetic control; GBN—gabapentin 100 mg·kg^−1^; S1.5 + M150—sildenafil 1.5 mg·kg^−1^ + metformin 150 mg·kg^−1^; S2.5 + M250—sildenafil 2.5 mg·kg^−1^ + metformin 250 mg·kg^−1^; S3 + M500—sildenafil 3 mg·kg^−1^ + metformin 500 mg·kg^−1^; * *p* < 0.05; ** *p* < 0.01; *** *p* < 0.001 vs. ND. ### *p* < 0.001 vs. D.

After the induction of diabetes with alloxan, changes in the blood glucose levels of the groups were observed (univariate ANOVA, F = 52.13, *p* < 0.0001, Figure 1A). Thus, we observed a significant increase in glycemia in all diabetic groups when compared to the non-diabetic control group (*p* < 0.001, Figure 1A).

After 7 days of treatment, significant changes in the blood glucose levels were noticed and maintained (univariate ANOVA, F = 88.91, *p* < 0.0001, Figure 1B). All diabetic groups had higher glycemia when compared to the non-diabetic group, but the S2.5 + M250 group and the S3 + M500 group showed a significant decrease when compared to the diabetic control group (*p* < 0.05, Figure 1B).

At day 14, blood glucose levels were significantly modified by the treatments (univariate ANOVA, F = 83.04, *p* < 0.0001, Figure 1C). We noticed significantly lower blood glucose values for all three combinations of sildenafil and metformin when compared to the diabetic control group (*p* < 0.001, Figure 1C). Moreover, the S2.5 + M250 and S3 + M500 groups demonstrated the best glycemic control with comparable effects of 55.28% and 55.70%, respectively, in reducing the blood glucose levels when compared to the initial values.

### 3.2. Evaluation of the Antihyperalgesic Effect

#### 3.2.1. Heat Hypersensitivity

**Figure 2:**
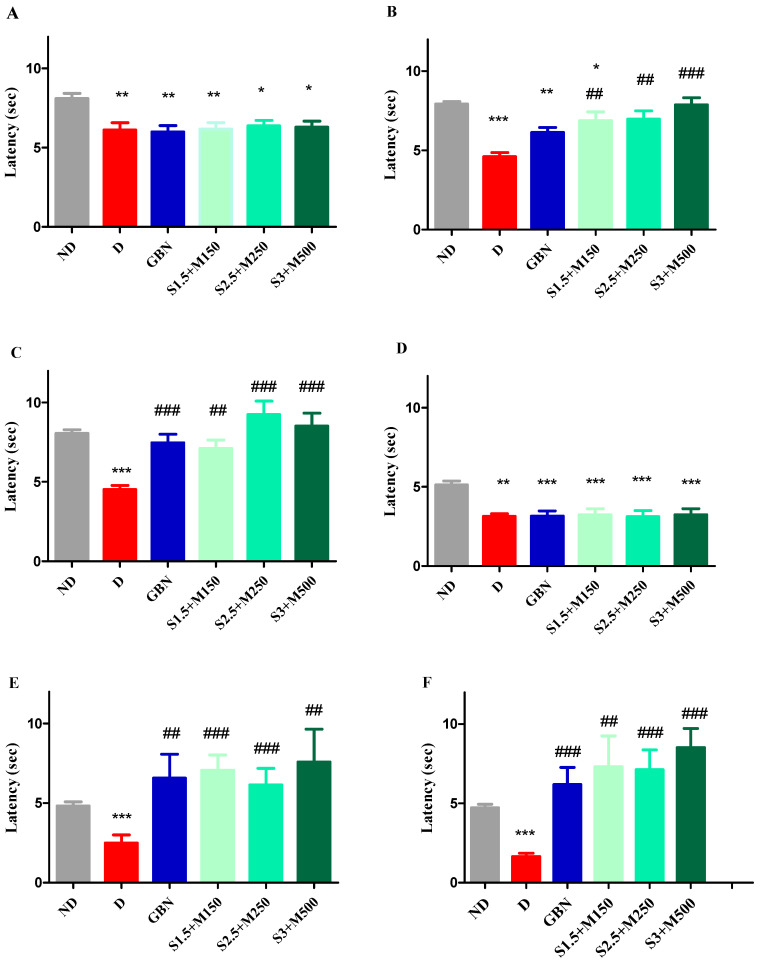
(**A**) Initial pain reaction latency in the hot-plate test (**B**). Pain reaction latency in the hot-plate test after 7 days. (**C**) Pain reaction latency in the hot-plate test after 14 days. (**D**) Initial pain reaction latency in the tail withdrawal test. (**E**) Pain reaction latency in the tail withdrawal test after 7 days. (**F**) Pain reaction latency in the tail withdrawal test after 14 days. Values are expressed as mean ± S.E.M. ND—non-diabetic control; D—diabetic control; GBN—gabapentin 100 mg·kg^−1^; S1.5 + M150—sildenafil 1.5 mg·kg^−1^ + metformin 150 mg·kg^−1^; S2.5 + M250—sildenafil 2.5 mg·kg^−1^ + metformin 250 mg·kg^−1^; S3 + M500—sildenafil 3 mg·kg^−1^ + metformin 500 mg·kg^−1^; * *p* < 0.05; ** *p* < 0.01; *** *p* < 0.001 vs. ND. ## *p* < 0.01; ### *p* < 0.001 vs. D.

In the hot-plate test, differences in pain sensitivity were observed between the non-diabetic control group and all diabetic groups before receiving the treatment (univariate ANOVA, F = 18.24, *p* < 0.0027, Figure 2A). Thus, all diabetic groups showed significantly lower licking times when compared to the non-diabetic control group before receiving the treatment (*p* < 0.05, Figure 2A).

Significant differences in thermal sensitivity were noticed after 7 consecutive days of treatment (univariate Anova, F = 42.43, *p* < 0.0001, Figure 2B), and also after 14 days (univariate ANOVA, F = 35.16, *p* < 0.0001, Figure 2C).

The group treated with gabapentin recorded an increase in pain reaction latencies after both 7 and 14 days of treatment when compared to the diabetic group, but a significant increase was observed only for the 14 day treatment periods (65, 25%, *p* < 0.001, Figure 2C).

All sildenafil–metformin combinations demonstrated an antihyperlagesic effect in the hot stimulus test. Thus, all three groups showed a significant decrease in pain sensitivity when compared to the diabetic group after 7 days of treatment, but the S3 + M500 group recorded the highest effect of 70.88% in increasing pain reaction latency (*p* < 0.001, Figure 2B). After 14 days of treatment, a significant increase in pain latencies was observed for all three drug combinations when compared to the diabetic control group, with a 104.64% increase for S2.5 + M250 and an 88.32% increase for S3 + M500 (*p* < 0.001, Figure 2C).

Furthermore, all three combinations produced a more pronounced antihyperalgesic effect than gabapentin after 7 consecutive days of treatment, while after 14 days of treatment, only S2.5 + M250 and S3 + M500 groups registered higher effects, with a 65.25% increase for gabapentin, 106.64% increase for S2.5 + M250, and 88.32% increase for S3 + M500, respectively, when compared to the diabetic control group. The increased intensity of the antihyperalgesic effect of combinations containing metformin, compared to those containing gabapentin, which is a reference substance in the treatment of neuropathic pain, is probably due to the reduction of blood glucose and implicitly of oxidative stress (Figure 2).

#### 3.2.2. Cold Hypersensitivity

Pain sensitivity to a cold stimulus showed differences between the non-diabetic control group and all diabetic groups prior to treatments (univariate ANOVA, F = 26.17, *p* < 0.0001, Figure 2D). Therefore, in the tail withdrawal test using water cooled to 10 °C, a similar significant higher pain sensitivity was observed for all diabetic groups when compared to the non-diabetic group at the beginning of the experiment before receiving the treatment (*p* < 0.05, Figure 2D).

Changes in pain reaction latencies were noticed after 7 days (univariate ANOVA, F = 26.33, *p* < 0.0001, Figure 2E) and after 14 days of treatments (univariate ANOVA, F = 37.05, *p* < 0.0001, Figure 2E).

Compared to the diabetic control, the group treated with gabapentin showed a significant increase in pain latency when compared to the diabetic control group both after 7 days, with a 2.63-fold increase (*p* < 0.01), and 14 days of treatment, with a 3.75-fold increase (*p* < 0.001) (Figure 2E).

In the case of sildenafil–metformin combinations, all three groups showed a significant decrease in pain sensitivity when compared to the diabetic control group in the two moments of determination (*p* < 0.05, Figure 1). After 7 days of treatment, S1.5 + M150 and S2.5 + M250 groups recorded significantly higher pain reaction latencies when compared to the diabetic control group (*p* < 0.001, Figure 2E). For comparison, S2.5 + M250 and S3 + M500 diabetic groups demonstrated the highest antihyperalgesic effect when compared to the diabetic control group after 14 days of treatment (*p* < 0.001, Figure 2F). Furthermore, after 7 and 14 days of treatment, the most pronounced effects were registered for the S3 + M500 group with a 3-fold and 5.17-fold decrease, respectively, in pain sensitivity when compared to the diabetic control group. Additionally, all three sildenafil–metformin combinations showed greater effects than gabapentin in decreasing pain sensitivity after 14 days of treatment, with a 3.75-fold decrease for the gabapentin group, 4.44-fold decrease for the S1.5 + M150 group, 4.33-fold decrease for the S2.5 + M250 group, and 5.17 decrease for the S3 + M500 group when compared to the diabetic control group (*p* < 0.05, Figure 2F). The results obtained in the cold stimulus test are consistent with those previously obtained in the hot-plate test and are supported by the increase in tail withdrawal latency from water cooled to 10 °C. Additionally, the greater intensity of the antihyperalgesic effect for the groups treated with the combinations containing metformin correlates with the improvement of the glycemic profile of the diabetic animals (Figure 2).

### 3.3. Biochemical Assay of Mouse Brain and Liver Homogenates

#### 3.3.1. Assessment of TNF-α and Il-6

We determined the level of tissue cytokines (TNF-α and IL-6) in the brain and liver, seeing as alloxan-induced diabetes is characterized by cytotoxicity in the pancreas, brain (neuronal degeneration and necrosis), kidney (nephrosis, nephritis), and liver (hepatitis) [40]. On the other hand, we evaluated if sildenafil–metformin combinations significantly influenced cytokine levels (TNF-α, IL 6) in tissues.

**Figure 3:**
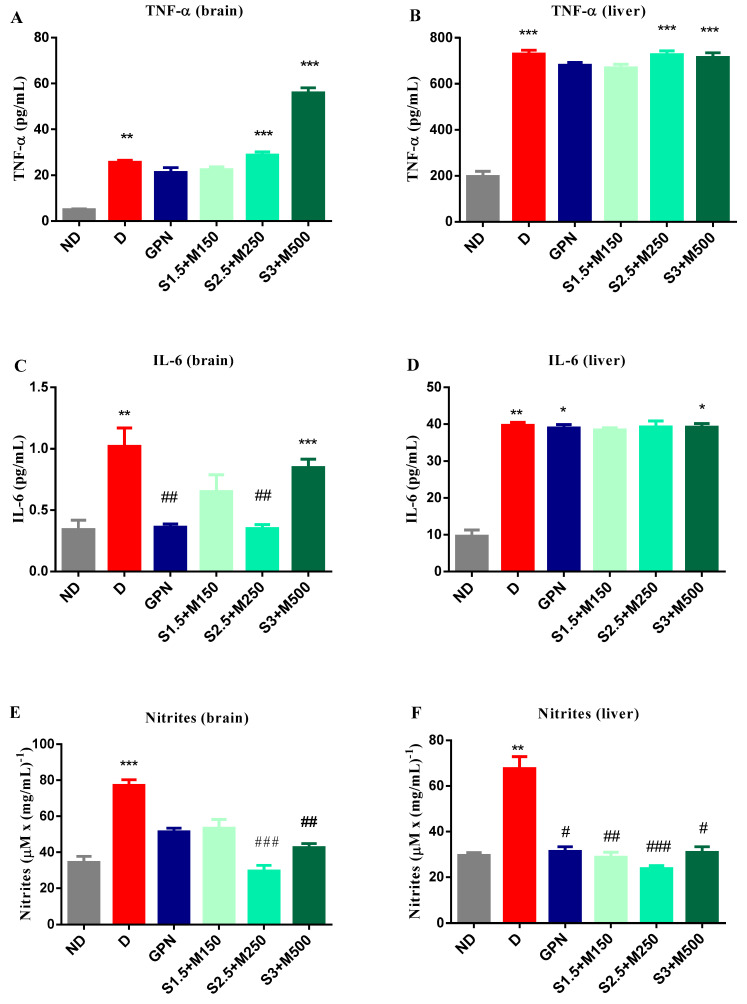
Results from biochemical assays of mouse brain and liver homogenates. (**A**) Variation of TNF-α in mouse brain tissues. (**B**) Variation TNF-α in mouse liver tissues. (**C**) Variation of IL-6 in mouse brain tissues. (**D**) Variation of IL-6 in mouse liver tissues. (**E**) Variation of nitrite/protein ratio in mouse brain tissues. (**F**) Variation of nitrite/protein ratio in mouse liver tissues. Values are expressed as mean ± S.E.M. ND—non-diabetic control; D—diabetic control; GBN—gabapentin 100 mg·kg^−1^; S1.5 + M150—sildenafil 1.5 mg·kg^−1^ + metformin 150 mg·kg^−1^; S2.5 + M250-sildenafil 2.5 mg·kg^−1^ + metformin 250 mg·kg^−1^; S3 + M500—sildenafil 3 mg·kg^−1^ + metformin 500 mg·kg^−1^; * *p* < 0.05; ** *p* < 0.01; *** *p* < 0.001 vs. ND. # *p* < 0.05; ## *p* < 0.01; ### *p* < 0.001 vs. D.

After 14 days of treatment, changes in the TNF-α levels were observed both in the brain (univariate ANOVA, F = 38.7, *p* < 0.0001, Figure 3A) and liver tissues (univariate ANOVA, F = 29.15, *p* < 0.0001, Figure 3B). Thus, the diabetic control group demonstrated 5.17-fold and 3.7-fold higher levels in brain and liver tissues, respectively, when compared to the non-diabetic group (*p* < 0.01, *p* < 0.001, Figure 3A,B).

Moreover, our research showed a significant increase in TNF-α levels in the diabetic control group, S2.5 + M250 and S3 + M500 groups in both brain and liver tissues when compared to the non-diabetic control group (*p* < 0.05, Figure 3). Contrastingly, the lowest increase was observed for the gabapentin group (4.26-fold higher in brain tissues and 3.46-fold higher in liver tissues) and the S1.5 + M150 group (4.51-fold higher in brain tissues and 3.41-fold higher in liver tissues) when compared to the non-diabetic group (Figure 3A,B).

The 14 days of treatment also resulted in differences concerning the IL-6 levels in the brain (univariate ANOVA, F = 29.6, *p* < 0.0001, Figure 3C) and liver tissues (univariate ANOVA, F = 13.59, *p* < 0.0185, Figure 3D). Compared to the non-diabetic control group, in the diabetic control group, we noticed 2.97-fold higher levels in the brain tissues and 4.12-fold higher levels in the liver tissues for IL-6 (*p* < 0.01, Figure 3C,D).

Interestingly, Il-6 levels showed a significant increase in the S3 + M500 group in the brain tissues when compared to the non-diabetic control group (*p* < 0.05, Figure 3C). In comparison, the gabapentin group and S2.5 + M250 group showed significantly lower levels of Il-6 in the brain tissues when compared to the diabetic control group, with a 65.62% decrease and 64.44% decrease, respectively (*p* < 0.01, Figure 3C).

In the liver tissues, we observed significantly higher levels of IL-6 for the gabapentin group and the S3 + M500 group when compared to the non-diabetic control group (*p* < 0.05, Figure 3D). A slight increase was observed for the S1.5 + M150 and the S2.5 + M250 groups versus the non-diabetic group (Figure 3D). Moreover, S1.5 + M150 showed the lowest increase (4.08-fold higher) when compared to the non-diabetic control group.

Figure 3A,B show that after 14 days of treatment since the initial induction of alloxan diabetes, the concentration of TNF-α increased in brain and liver tissues in all groups included in this study. IL-6 concentration also increased in these tissues (Figure 3C,D). Although many studies demonstrated that sildenafil and metformin decrease the concentration of TNFα in blood, our experiment shows that the administration of metformin-sildenafil combinations does not reduce the concentration of the cytokine in brain and liver tissue.

#### 3.3.2. Griess Assessment of NOS Activity

An increase in TNF-α release in the brain, induced by oxidative stress after alloxan administration, is preceded by the activation of the TNF-α-converting enzyme (TACE) [41], and, subsequently, it stimulates iNOS gene expression via the factor nuclear factor jB (NF-jB) [42]. Inducible nitric oxide synthase (NOS-2, or iNOS) is involved in the cellular toxicity of many systems [43]. Thus, we determined the brain and liver concentration of nitrites formed under the action of iNOS in animals treated with different combinations of sildenafil and metformin.

Nitrites levels exhibited differences after 14 days of treatment both in brain tissues (univariate ANOVA, F = 35.43, *p* < 0.0001, Figure 3E) and liver tissues (univariate ANOVA, F = 27.8, *p* < 0.0001, Figure 3F). Our study showed a significant increase in nitrites for the diabetic group in both brain and liver tissues when compared to the non-diabetic group (*p* < 0.05, Figure 3). The increase was similar in brain tissues and liver tissues, i.e., 2.24-fold vs. 2.28-fold higher for the diabetic control group.

In the brain tissues, a significant decrease in nitrites was noticed for the combinations S2.5 + M250 and S3 + M500 when compared to the diabetic control group, with a 61.65% decreae and 44.77% decrease, respectively (*p* < 0.05, Figure 3E). On the other hand, regarding the liver tissues, the diabetic group that received gabapentin and the groups treated with all three combinations of sildenafil and metformin recorded significantly lower increases when compared to the diabetic control group, with a 64.8% decrease for the S2.5 + M250 group, a 57.35% decrease for the S1.5 + M150 group, and a 54.35% decrease for the S3 + M500 group (*p* < 0.05, Figure 3F).

The decrease in brain nitrites concentration correlates with the decrease in pain sensitivity, expressed by the increase in pain reaction latency of animals treated with gabapentin and sildenafil–metformin combinations in the hot stimulus test (Figure 3) and in the cold stimulus test (Figure 3).

## 4. Discussion

The main purpose of our study was to investigate the antihyperalgesic potential of the sildenafil–metformin combination in diabetic neuropathy and the influence of this combination on inflammatory and redox status. Previously, we demonstrated that both drugs can reduce thermal hyperalgesia after 14 days of treatment [26], and we set out to determine if their combined action manifests the same effect; our study, to the best of our knowledge, is the first of its kind.

One of the common complications of diabetes is erectile dysfunction (ED). A meta-analysis conducted by Kouidrat et al. showed that the incidence of ED in diabetic patients was 52.5% and its prevalence was approximately 3.5 times higher than in non-diabetic patients [44]. The efficacy, safety, and improvement of the quality of life of patients with type-2 diabetes and ED using sildenafil is well established [45,46].

The importance of metformin in the treatment of type-2 diabetes is well established by the guidelines of the American Diabetes Association and the European Association for the Study of Diabetes [47], being recommended as a first-line pharmacological therapy. Taking into account that these two drugs can be frequently prescribed to maintain glycemic control and to correct ED in type-2 diabetic patients, we aimed to investigate whether the combination of the two substances can correct diabetic neuropathy, a frequent complication of this disease.

In our study, we induced DN by the administration of single or multiple doses of alloxan. Inducing diabetes via alloxan is one of the most used methods to investigate several aspects of diabetes and its complications [48]. Among the advantages of this method are its low cost, high and easy reproducibility, stable model, and high percentage of diabetic animals [49].

Alloxan has a very high structural similarity to glucose and, due to its toxicity to the β-cells of the pancreas, it stimulates a type-1 form of diabetes when administered to animals [50,51]. Literature data suggest that one of the mechanisms contributing to the development of hyperalgesia in rodents involves oxidative stress induced by elevated glucose levels [52]. According to this, our research demonstrated that the diabetic control group had a significant increase in pain sensitivity when compared to the non-diabetic control group in both hot and cold stimulus tests during the 14 days of the experiment.

After the onset of alloxan-induced diabetes, significant high blood glucose levels (≥180 mg/dL) were maintained during the experiment in diabetic groups versus the non-diabetic group. The sildenafil–metformin combinations groups recorded a significant decrease in blood glucose levels at the end of the experiment when compared to the diabetic control group due to the hypoglycemic action of metformin.

Recent studies suggested that sildenafil could be a potential therapeutic option in inflammatory and neurodegenerative diseases through the accumulation of cGMP (cyclic guanosine monophosphate). cGMP is involved in the regulation of vascular function, axon conduction, and synaptic plasticity. It is produced by soluble cytoplasmic guanylate cyclase, and phosphodiesterase 5 (PDE5) is the enzyme involved in its hydrolysis [53,54]. PDE5 expression increases due to hyperglycemia, and following the inhibition of the enzyme by sildenafil, an increase in cGMP levels occurs with a consequent improvement of the symptoms of DN [55]. Other experimental research suggests that treatment of diabetic peripheral neuropathy may include sildenafil as it improves circulation in the sciatic nerve [53]. Moreover, clinical data support the antihyperalgesic effect of sildenafil administered to diabetic patients for the treatment of erectile dysfunction [55].

Bandera et al. experimentally showed that sildenafil reduces flap necrosis by growing the secretion of fibroblast growth factor and vascular endothelial growth factor [56]. Furthermore, research on 36-week-old diabetic mice showed that the administration of sildenafil significantly increased the number of functional blood vessels and regional blood flow in the sciatic nerve as well as the density of intra-epidermal nerve fibers in the skin and myelinated axons in the sciatic nerve. In addition, sildenafil treatment significantly improved motor and sensory conduction velocities in the sciatic nerve and sensitivity to peripheral thermal stimulus and reversed the downregulation of angiopoietin 1 expression in the mouse dermal endothelial cell cultures caused by high glucose levels [22]. Vascular dysfunction leads to sciatic nerve damage in diabetic neuropathy. Angiopoietins and their receptor, Tie-2, participate in vascular stabilization and neurite outgrowth in cultured dorsal root ganglion neurons [57].

Metformin has multiple mechanisms of action, including AMPK activation, inhibition of mitochondrial respiration, increasing insulin sensitivity, and antagonizing the effects of glucagon [58].

Price et al. suggested that drugs that modulate the activity of AMPK may be a new alternative for chronic pain therapy. A diversity of cellular processes are regulated by AMPK, including mitochondrial metabolism, protein translation, and the activity of other kinases, many of which are implicated in the development of neuropathic pain [59,60]. Metformin has a demonstrated ability to activate AMPK and consequently can be tested for its antihyperalgesic effect in various pain models [61].

The use of metformin after induction of nerve injury was reported to reverse tactile allodynia in mice and attenuate tactile allodynia induced by spinal nerve ligation in rats [59]. Martin-Montalvo et al. investigated the pleiotropic effects of metformin by administering it in the diet of middle-aged male mice. The research concluded that the administration of metformin to non-diabetic animals mimics the benefits of caloric restriction: increased physical performance, increased tissue sensitivity to insulin, and decreased total and LDL cholesterol. These beneficial effects of metformin are the results of its molecular actions, AMPK activation, and antioxidant protection, resulting in the reduction of both oxidative stress and inflammation [62].

In addition, the antihyperalgesic effect of metformin in diabetic mice is also supported by a recent study that demonstrated that the antihyperglycemic agent decreases tolerance to morphine following chronic administration. Thus, metformin (10 mg·kg^−1^) was administered to mice 45 min before each morphine administration. The evaluation of the analgesic action was carried out employing tail flick and hot plate tests. Consequently, chronic administration of metformin significantly increased analgesic latency in both tests, specifically on days 3 and 5, when compared to the group that received only morphine in both tests. The results are based on the fact that chronic and acute administration of metformin significantly decreased the level of nitric oxide. Moreover, by activating the AMPK pathway, metformin inhibits the activity of mTOR (mammalian target of rapamycin) whose increase contributes to the emergence of opioid tolerance [63]. Furthermore, a study showed that metformin can reduce mechanical allodynia by down-regulating NF-κB (nuclear factor kappa B) expression [63].

In accordance with these results, we investigated the antihyperlagesic effect of sildenafil–metformin combination in different doses. The doses of sildenafil and metformin used in our study were chosen based on previous experimental studies in animal models of pain. Bezzera et al. evaluated the antinociceptive effect of sildenafil and sympatholytic agents (propranolol, atenolol, prazosin, and clonidine) in the writhing test in mice. In this test, sildenafil was administered in doses of 2.5 and 5 mg·kg^−1^ [64]. Naveen et al. showed that sildenafil in a dose of 2 mg·kg^−1^ i.p. potentiates the analgesic effect of morphine in two tests: carrageenan-induced hyperalgesia in rats and the acetic-acid-induced writhing test in mice [65]. Patil et al. evaluated the modulatory effect of cyclooxygenase inhibitors on the sildenafil-induced antinociception writhing assay test and carrageenan-induced hyperalgesia test in mice and rats and administered sildenafil in doses of 1–2 mg·kg^−1^ i.p [66]. Regarding the doses of metformin used in our study, Das et al. administered metformin in a dose of 200 mg·kg^−1^ i.p. in a mice model of complex regional pain. The same authors highlighted the antihyperalgesic effect of metformin administered in a dose of 200 mg·kg^−1^ orally in a mouse model of postoperative pain [67]. Augusto et al. showed that the administration of single or repeated doses of metformin (250, 500, or 1000 mg·kg^−1^) inhibits mechanical allodynia induced by chronic constriction injury [68]. Furthermore, in a previous study, we determined the dose–antihyperalgesic effect relationship for these two substances when administered as a monotherapy: sildenafil: 1.5; 2.5; 3 mg·kg^−1^ orally, metformin 50; 100; 150 mg·kg^−1^ orally, and gabapentin 150; 250; 500 mg·kg^−1^ orally [26]. As the present study aimed to determine whether there is a synergistic effect for the treatment of neuropathic pain between sildenafil and metformin, we combined these two substances in the same doses as used alone. We chose the oral route because both substances have good bioavailability, and in chronic treatment the oral route is preferred.

In our study, in the hot stimulus test, all three combinations showed a significant decrease in pain sensitivity when compared to the diabetic control group both after 7 and 14 days of treatment. On the seventh day of the experiment, the S3 + M500 combination demonstrated a better antihyperalgesic effect, while after the fourteenth day of treatment, the S2.5 + M250 group showed a higher increase in pain reaction latency.

In the withdrawal tail test, the antihyperalgesic effect of the three sildenafil–metformin combinations observed in the hot-plate test was sustained, with the S3 + M500 recording a lower increase in pain sensitivity when compared to the diabetic control group after 14 days of treatment.

As a first-line therapy in DN, gabapentin has demonstrated its antihyperalgesic effect in several preclinical studies. Thus, Back et al. showed that different doses of gabapentin (30, 100, and 300 mg·kg^−1^) ameliorate mechanical, hot, and cold allodynia in partial tail-nerve-injury-induced neuropathic pain [69]. Zbârcea et al. demonstrated that treatment with gabapentin reduced allodynia in paclitaxel-induced neuropathy in rats [70]. Another study highlighted the benefits of 50 mg·kg^−1^ gabapentin i.p., as it reduced cold and mechanical allodynia in a saphenous partial ligation animal model of neuropathic pain [71]. Moreover, Xiao et al. indicated that gabapentin in multiple doses of 100 mg·kg^−1^ i.p. significantly decreased mechanical allodynia and hyperalgesia in paclitaxel and vincristine-induced neuropathic pain [72]. In DN animal models, oral gabapentin (50 mg·kg^−1^) for 5 days alleviated mechanical allodynia [73] and doses of 10, 20, or 40 mg·kg^−1^ gabapentin p.o. showed an increase in the withdrawal threshold in the von Frey test and prolonged reaction time in the hot-plate test [74]. Our study supports these results, demonstrating the antihyperalgesic effect of gabapentin in DN. Thus, the gabapentin group showed significantly higher pain reaction latency after 14 days of treatment in the hot-plate test, while a significantly lower pain sensitivity was observed both after 7 and 14 days of treatment when compared to the diabetic group in the cold stimulus test.

Damage to peripheral nerves and nerve roots results in neuro-inflammation and neuro-immune activation, indicating that nitric oxide (NO) and pro-inflammatory cytokines play a significant role in the development of neuropathic pain. Studies carried out on animal models, as well as clinical ones, have demonstrated the role of cytokines in inducing and maintaining pain [75,76,77]. Nerve injury results in the dedifferentiation of Schwann cells and delivery of analgesic mediators such as pro-inflammatory cytokines, nerve growth factor, prostaglandin E2 (PGE2), and adenosine triphosphate (ATP). This release of mediators leads to an elevated inflammatory response in the injured nerve and consequently plays a role in the occurrence of neuropathic pain [75,78].

TNF-α is a pro-inflammatory cytokine whose involvement in neuropathy is more obvious than those of IL-6 or C-reactive protein [79]. Several studies in animal models of neuropathic pain have demonstrated that TNF-α plays an essential role both in peripheral and central levels of sensitization [80]. The endogenous production of TNF-α increases at the level of microvascular and neuronal tissues in the case of chronic hyperglycemia. This leads to increased microvascular permeability, hypercoagulability, and nerve damage, which contribute to the development of DN [81]. Phosphorylation of extracellular regulated kinase and mitogen-activated protein kinase p38 mediates the action of TNF-α on neurons [82]. Mechanical allodynia may be mediated by p38 mitogen-activated protein kinase phosphorylation via the modulation of tetrodotoxin-resistant Na channels [83]. Additionally, NF-κB is activated by TNF-α to initiate NOS (NO synthase) and NO production, with NO being a pain neurotransmitter [84]. TNF-α effect on the production of phospholipase A2, involved in regulating the availability of arachidonic acid and cyclooxygenase, leads to altered synthesis of PGE2, prostaglandin I2 (PGI2), and prostaglandin F2α (PGF2α) [85,86]. In addition, TNF-α blocks endothelium-dependent relaxation and stimulates mesangial cell contraction in a time and dose-dependent manner [87]. Afshari et al. showed that metformin decreases the TNF-α levels in the spinal cord and inhibits its receptors, indicating its neuroprotective and anti-inflammatory actions in neuropathy in a spinal cord injury animal model [88]. Another study demonstrated that metformin reduced the mRNA (messenger ribonucleic acid) expression of TNF-α induced by morphine by decreasing spinal microglial activation [89]. an increase in TNF-α release in the brain, induced by oxidative stress after alloxan administration, is preceded by the activation of the TNF-α converting enzyme (TACE) [41], and, subsequently, it stimulates iNOS gene expression via nuclear factor jB (NF-jB) [42]. Moreover, other reports have indicated that the anti-inflammatory effect of metformin involves reduced TNF-α production in macrophages [90]. Additionally, type-2 diabetic patients presented lower levels of TNF-α after treatment with metformin [91]. Additionally, sildenafil reduces TNF-α production in microglial cells by inhibition of NF-κB and MAPKs (mitogen-activated protein kinases), demonstrating its anti-inflammatory action [92].

In our study, we observed that TNF-α levels were significantly increased in the diabetic control group and the S2.5 + M250 and S3 + M500 groups both in the brain and liver. On the other hand, lower increases were demonstrated for the gabapentin group and the S1.5 + M150 when compared to the non-diabetic group.

The present research highlighted that the cytotoxicity of alloxan in rodents is also mediated by the increase in the concentration of pro-inflammatory cytokines in the brain and liver tissue, a phenomenon that is not reversed by the administration of sildenafil and metformin. Preclinical studies showed that in diabetic animals there is an increase in TNF-α concentration in serum, and this in turn induces an increase in the expression of the sodium channel NaV1.7, which results in the sensitization of nerve endings and implicitly the development of diabetic neuropathy. To elucidate this mechanism, dorsal root ganglion (DRG) neurons were exposed (for 6 h) to concentrations of TNF-α equivalent to those measured in streptozotocin-induced rats that developed hyperalgesia. The results showed that TNF-α induced an increase in Na+ current density in dorsal root ganglion neurons. The pain hypersensitivity recorded in diabetic animals may be due to the activation of the NaV1.7 sodium channel [93].

Another pro-inflammatory cytokine that exhibits neurotrophic activity, IL-6, contributes to neuronal development and predicts the progression of diabetes, and is also a sensitive marker for diabetic nephropathy [87]. In preclinical studies, IL-6 improved the appearance of small and large nerve fibers [94]. IL-6 was associated with increased nerve blood flow through a mechanism involving endothelium-derived hyperpolarizing factor. Increases in IL-6 levels were observed in the progression of nerve degeneration in DN. This cytokine alters glial cells and neurons, which results in starting the pathological process of DN [95]. Zheng et al. showed that serum TNF-α and IL-6 were significantly increased in patients with type-2 diabetes [96]. In a study conducted by Magrinelli et al., serum IL-6 was increased in 44% of diabetic patients, being inversely correlated with sensory nerve action potentials and compound muscle action potentials. The authors believed that IL-6 induces axonal degradation in peripheral nerves [97]. A study showed that metformin decreases mRNA expression of IL-6 induced by morphine, indicating that metformin reduces neuroinflammation through AMPK-mediated signaling [89]. Moreover, another study showed metformin could also reduce IL-6 production by decreasing NF-κB translocation [74]. In addition, sildenafil decreased IL-6 levels and enhanced endothelial function in diabetic patients [98,99].

Our research showed that the groups treated with gabapentin and S2.5 + M250 combination showed significant decreases in IL-6 levels in brain tissues when compared to the diabetic control group. In comparison, in liver tissues, lower increases were observed for S1.5 + M150 and S2.5 + M250 combinations, respectively.

NO (nitric oxide) is also implicated in the pathogenesis of DN through the upregulation of inducible nitric oxide synthase (iNOS) resulting from chronic inflammation. NO inhibits platelet aggregation and smooth muscle proliferation [81]. Several studies showed a reciprocal interaction between the pathways of NO and prostaglandins and demonstrated that high levels of NO increase the progression of DN [100,101,102]. Therefore, NO intervenes directly in the expression of COX and implicitly in the biosynthesis of PGs (prostaglandins). Instead, NO biosynthesis is influenced by the COX isoforms through arachidonic acid and the metabolites produced. Through post-transcriptional and translational processes that result in high macrophage PG production, NO influences COX-2 enzyme activities [103]. iNOS is deactivated by blocking the NF-κB signal following the release of NO by constitutive NOS. NF-κB signaling is one of the signaling pathways for iNOS expression through extracellular mediators, such as TNF-α and endotoxin [104]. By inhibiting NF-κB, NO manifests its inhibitory role in COX-2, with NF-κB acting as a potent inducer of COX-2. In peripheral tissues, several cell types produce NO after inflammation has set in. Once expressed, iNOS induces high amounts of NO for an extended period [105]. Metformin demonstrated its capacity to reduce the expression of iNOS [106] and NO production by down-regulation of NF-κB translocation [89], while sildenafil suppresses the expression of iNOS by decreasing its mRNA and protein levels [92].

In our study, after 14 days of treatment, nitrites levels were significantly decreased for S2.5 + M250 and S3 + M500 combinations in brain tissues when compared to the diabetic control group, while all diabetic groups that received treatment with gabapentin and the three sildenafil–metformin combinations demonstrated significant decreases in nitrites levels in liver tissues when compared to the diabetic control group, which suggest a lower NOS activity.

Overall, our findings demonstrated for the first time, that a sildenafil–metformin combination could effectively relieve the symptoms of diabetic neuropathy. The advantage of this association can be attributed to the different mechanisms of action of these two drugs, which may potentiate their effects. Besides these, we noticed that the combination of the lowest doses used of sildenafil and metformin showed comparable effects to the other two combinations in alleviating pain, which suggests that lower doses of both drugs can be used to prevent dose-dependent side-effects. Our results are in line with other studies that suggest pharmacotherapeutic approaches with a combination of two drugs could be more efficient in treating diabetic neuropathy [107]. Furthermore, the sildenafil–metformin combination reduced the production of IL-6 and concentration of nitrites, which indicates its ability to reduce inflammation involved in the progression of diabetic neuropathy.

The main limitations of our study were the relatively short duration of the treatment (14 days), imposed by the increased toxicity of alloxan, which does not permit the assessment of pro-inflammatory cytokines in the conditions of installation of a chronic pathology and its complications and the high variability of pain sensitivity [108].

## 5. Conclusions

Our study aimed to investigate, for the first time, the synergistic effect of a sildenafil–metformin combination, administered in different doses, on pain sensitivity in an alloxan-induced diabetic neuropathy model. The combination of these two substances, in a dose-dependent manner, increased the pain reaction latency of diabetic animals. Moreover, all sildenafil–metformin combinations significantly reduced the concentration of nitrites in the brain and liver, which are final products formed under the action of iNOS and are responsible for cellular toxicity at various levels, including at the neuronal level, when compared to the diabetic control group. The decrease in pain sensitivity induced by the sildenafil–metformin combinations through various mechanisms correlates with the reduction of iNOS activity, whose expression is increased by the high concentrations of TNFα and IL-6 detected in the brain and liver. Considering these findings, sildenafil–metformin combinations may prove to be proper alternative options in ameliorating pain in DN.

## Data Availability

All data generated or analyzed during this study are included in this published article.
